# General Features and Novel Gene Signatures That Identify Epstein-Barr Virus-Associated Epithelial Cancers

**DOI:** 10.3390/cancers14010031

**Published:** 2021-12-22

**Authors:** Chukkris Heawchaiyaphum, Chamsai Pientong, Hironori Yoshiyama, Hisashi Iizasa, Watcharapong Panthong, Tipaya Ekalaksananan

**Affiliations:** 1 Department of Microbiology, Faculty of Medicine, Khon Kaen University, Khon Kaen 40002, Thailand; jukkris.003@gmail.com (C.H.); chapie@kku.ac.th (C.P.); watchara.p@kkumail.com (W.P.); 2 HPV&EBV and Carcinogenesis (HEC) Research Group, Faculty of Medicine, Khon Kaen University, Khon Kaen 40002, Thailand; 3 Department of Microbiology, Shimane University Faculty of Medicine, Izumo 693-8501, Japan; yosiyama@med.shimane-u.ac.jp (H.Y.); iizasah@med.shimane-u.ac.jp (H.I.)

**Keywords:** transcriptomics, EBV, NPC, EBVaGC, OSCC, TMC8, SLA29A9, IL6/JAK/STAT3 signaling, TNF-α/NF-κB signaling

## Abstract

**Simple Summary:**

Nasopharyngeal carcinoma (NPC), Epstein-Barr virus (EBV)-associated gastric carcinoma (EBVaGC), and oral squamous cell carcinoma (OSCC) are epithelial cancers that are associated with EBV infection. However, the gene signatures and common hallmarks associated with EBV infection in EBV-associated epithelial cancers (EBVaCAs) have not been fully elucidated. Here, we performed a panel of transcriptome analyses to identify the gene signatures and common hallmarks of these EBVaCAs. Based on the changes in the expression levels of genes in EBV-infected cell lines and tumor tissues, we identified two upregulated genes, SLC26A9 and TMC8, as gene signatures for EBVaCAs. In addition, SLC26A9 and TMC8 are differentially expressed genes (DEGs) in EBV-infected cells, and their expression is highly correlated with the stimulation of genes involved in numerous biological processes and pathways, such as IL6/JAK/STAT3 and TNF-α/NF-κB signaling pathways. Here, we propose SLC26A9 and TMC8 as novel gene signatures. In addition, we propose IL6/JAK/STAT3 and TNF-α/NF-κB signaling pathways as common hallmarks of EBVaCAs.

**Abstract:**

Epstein-Barr virus (EBV) is associated with various types of human malignancies, including nasopharyngeal carcinoma (NPC), EBV-associated gastric carcinoma (EBVaGC), and oral squamous cell carcinoma (OSCC). The present study aimed to identify gene signatures and common signaling pathways that can be used to predict the prognosis of EBV-associated epithelial cancers (EBVaCAs) by performing an integrated bioinformatics analysis of cell lines and tumor tissues. We identified 12 differentially expressed genes (DEGs) in the EBVaCA cell lines. Among them, only four DEGs, including BAMBI, SLC26A9, SGPP2, and TMC8, were significantly upregulated. However, SLC26A9 and TMC8, but not BAMBI and SGPP2, were significantly upregulated in EBV-positive tumor tissues compared to EBV-negative tumor tissues. Next, we identified IL6/JAK/STAT3 and TNF-α/NF-κB signaling pathways as common hallmarks of EBVaCAs. The expression of key genes related to the two hallmarks was upregulated in both EBV-infected cell lines and EBV-positive tumor tissues. These results suggest that SLC26A9 and TMC8 might be gene signatures that can effectively predict the prognosis of EBVaCAs and provide new insights into the molecular mechanisms of EBV-driven epithelial cancers.

## 1. Introduction

Epstein-Barr virus (EBV) is a ubiquitous gamma-herpesvirus that infects more than 90% of the world’s population. EBV infection establishes lifelong persistence in the human body by infecting various types of cells, including B lymphocytes, T cells, natural killer cells, and epithelial cells. Primary EBV infection in childhood usually shows few or no symptoms, whereas persistent EBV infection often leads to lymphocytic or epithelial malignancies, such as nasopharyngeal carcinoma (NPC) and gastric carcinomas (EBV-associated gastric carcinoma [EBVaGC]) [1,2,3]. Recently, the number of studies describing the association of EBV with oral squamous cell carcinoma (OSCC) has been increasing [4,5,6,7].

Histological examination of lesion biopsy is the standard method for determining the clinical stage, prognosis, and treatment of cancer, including NPC, gastric cancer (GC), and OSCC [8,9,10]. However, this procedure is not suitable for the early diagnosis or screening of patients with malignancies because the procedure is invasive and painful. Instead, detection of serum EBV DNA has recently been used as a biomarker of EBV-associated epithelial cancers (EBVaCAs), such as NPC and GC [11,12,13,14]. However, new biomarkers that reflect tumor heterogeneity should be investigated to evaluate their clinical usefulness and to develop personalized therapies. Therefore, exploring signature genes and key molecular mechanisms is critical for the early diagnosis and prognostic evaluation of patients with EBVaCAs.

Recently developed transcriptomic analysis can effectively identify changes in gene expression profiles under certain pathophysiological conditions. Thus, this analysis is useful for investigating the underlying molecular mechanisms and biological consequences of altered gene expression [15].

A growing body of evidence indicates that transcriptomic analysis has great potential for predicting clinical outcomes and therapeutic responses. Using this approach, various genes were identified as gene signatures that have significant clinical implications for the prognosis prediction of disease progression and outcomes [16,17,18,19]. The FBXO17 and PPARGC1A genes were identified as gene signatures for colon cancer and could be useful in predicting the survival outcome and establishing the appropriate treatment strategy [20]. The HDAC1, BIRC5, SPP1, STC2, and NR6A1 genes have been proposed as prognostic models of immune genes in hepatocellular carcinoma [21]. The nine glycolysis-related genes were identified as predictive signatures and may function as independent and important risk factors for ovarian cancer [22]. In addition, several long non-coding RNAs have been identified as predictors of prognosis and immunotherapeutic response in several types of cancers, including GC, colorectal cancer, and hepatocellular carcinoma [23,24,25].

It is helpful in screening tumor markers to improve the early clinical diagnostic skills and ultimate therapeutic effects. The current study was the first to identify and validate the gene signature and common hallmarks of EBVaCAs by combining experimental results and bioinformatics analysis of publicly available transcriptomic data.

## 2. Materials and Methods

### 2.1. Cell Lines

NPC cell lines (HONE1 and HONE1-EBV cells) and GC cell lines (AGS and AGS-EBV cell lines) were cultured in Roswell Park Memorial Institute 1640 Medium (Sigma, St. Louis, MO, USA). EBV-negative OSCC cell lines, SCC25 and HSC1 cell lines, (kindly provided by Dr. Tohru Kiyono, National Cancer Center (NCC), Chiba, Japan) and previously established EBV-positive OSCC cell lines, SCC25-EBV and HSC1-EBV cell lines [7], were maintained in Dulbecco’s Modified Eagle Medium/F12 (Sigma, St. Louis, MO, USA). All cell lines were supplemented with 10% fetal bovine serum (Gibco, Breda, The Netherlands) and penicillin-streptomycin solution. The cells were cultured at 37 °C in a 5% CO_2_ incubator.

### 2.2. DNA Microarray

Total RNA was extracted from HSC1, HSC1-EBV, SCC25, and SCC25-EBV cells using ISOGEN reagent (Nippon Gene, Tokyo, Japan) according to the manufacturer’s instructions. RNA was first quantified and qualified using a Qubit^®^ 2.0 Fluorometer and 2100 Bioanalyzer. The RNA samples were scanned using an Agilent G4900DA SureScan Microarray Scanner System. Genes with a fold-change greater than 1.5 were selected for further analysis. The microarray analysis service was provided by DNA Chip Research Inc. (Tokyo, Japan).

### 2.3. Collection and Processing of Publicly Available Data

The RNA sequencing data of NPC and GC cell lines and tumor tissues were obtained from the Gene Expression Omnibus (GEO) database. The RNA sequencing datasets, including GSE60873 [26], GSE147512, GSE54174 [27], GSE51575 [26] GSE102349 [28], GSE68799, GSE74956 [29], and GSE118719 [30], were downloaded and analyzed in this study. The raw RNA sequencing reads were first aligned to a reference human genome (hg38) using Spliced Transcript Alignment to a Reference aligner v2.7.3a. In addition, the unmapped reads were used to determine the EBV infection status by mapping with the reference genome of EBV (EBV_Akata_inverted, AJ507799.2). EBV-positive cases were selected for read counts of more than 300 read counts [31].

The Galaxy platform was used (https://usegalaxy.org/, accessed on 17 February 2021) to examine the expression of host genes. The quality of sequences was assessed using FastQC (Galaxy Version 0.72+ galaxy1). The raw sequencing reads that passed the quality control (QC) were aligned to the reference human genome (hg38) using HISAT2 (Galaxy Version 2.2.1+ galaxy0). To examine the gene expression from RNA sequencing data, the BAM files generated by HISAT2 were introduced to featureCounts (Galaxy Version 2.0.1+ galaxy1). The differentially expressed genes (DEGs) were determined from the count matrix using DESeq2 (Galaxy Version 2.11.40.6+ galaxy2). The DEGs with the *p*-value adjustment greater than 0.05 and log2(fold change) equal or greater than 2 were selected for further analysis.

### 2.4. Data Analysis

To screen the DEGs of EBVaCAs, a Venn diagram was constructed to show unique or shared DEGs in EBV-associated cancer cell lines using the jvenn online tool (http://bioinfo.genotoul.fr/jvenn, accessed on 11 March 2021). The functional annotation (GO) of DEGs was performed using the Database for Annotation, Visualization, and Integrated Discovery v6.8 web tool (https://david.ncifcrf.gov/, accessed on 28 June 2021) to analyze the target functional activities of DEGs. GO terms with a *p*-value of >0.05 were considered significant, and the top 10 categories were presented. Kyoto Encyclopedia of Genes and Genomes (KEGG) pathway analysis was performed to examine the functional pathways associated with DEGs. The top 10 pathways were presented. The volcano plot and bubble plot were generated by the “ggplot2” R package.

To examine the common molecular mechanism by which EBV promotes carcinogenesis of EBV-associated cancers, the Gene Set Enrichment Analysis (GSEA; version 4.0.3), a computational method to sequence the gene according to the expression level and determine if the gene sets defined initially are statistically significant. The computational annotated hallmark gene set from the Molecular Signatures Database was used. The gene sets showing a *p*-value less than 0.05 were considered significant hallmarks.

In addition, to determine the possibility of using candidate gene signatures as biomarkers for EBV-associated malignancies, the CCLE database was used to analyze the association of the candidate gene signature expression with the EBV-associated cancer lines, including lymphoma and epithelial cancers.

### 2.5. Verification of Candidate Genes Expression in Cell Lines and Tumor Tissues

Total RNA was extracted using TRIzol™ reagent (Invitrogen, Carlsbad, CA, USA) according to the manufacturer’s instructions, and 1 µg of RNA was used to synthesize cDNA using SuperScript^®^ III Reverse Transcriptase (Invitrogen, Carlsbad, CA, USA) according to the manufacturer’s instructions. Gene expression was quantified by quantitative reverse transcription-polymerase chain reaction (qRT-PCR) assay using SsoAdvanced^TM^ SYBR^®^ Green Supermix (Bio-Rad, Hercules, CA, USA) in the QuantStudio 6 Flex Real-Time PCR System (Applied Biosystems, Foster City, CA, USA). Glyceraldehyde 3-phosphate dehydrogenase (GAPDH) was used as an internal control. The relative mRNA expression levels were quantified using the 2^−^^ΔΔCT^ method. The primers used in this study are listed in Table 1.

### 2.6. Statistical Analysis

GraphPad Prism (GraphPad Software Inc., San Diego, CA, USA) was used to analyze data. The Mann-Whitney test in Graphpad was used to analyze the difference of gene expression in cell lines and tumor tissues between EBV-positive and EBV-negative cells and tumors. The significance of differentially expressed genes and signaling pathways was tested by the approaches used in this study, Galaxy platform and GSEA, respectively. All experiments were performed in duplicates for two-independent times. Statistical significance was set at *p* < 0.05.

## 3. Results

### 3.1. Identification of Differentially Expressed Genes in EBVaCAs

We analyzed the gene expression profiles of three EBVaCAs: OSCC, NPC, and GC. Microarray analysis was performed on EBV-positive OSCC cell lines, HSC1-EBV cells and SCC25-EBV cells, and their counterpart EBV-negative OSCC cell lines, HSC1 cells and SCC25 cells, respectively [7]. In addition, the RNA sequencing data of EBV-positive and EBV-negative cell lines derived from NPC or GC were retrieved from the GEO dataset.

**Figure 1:**
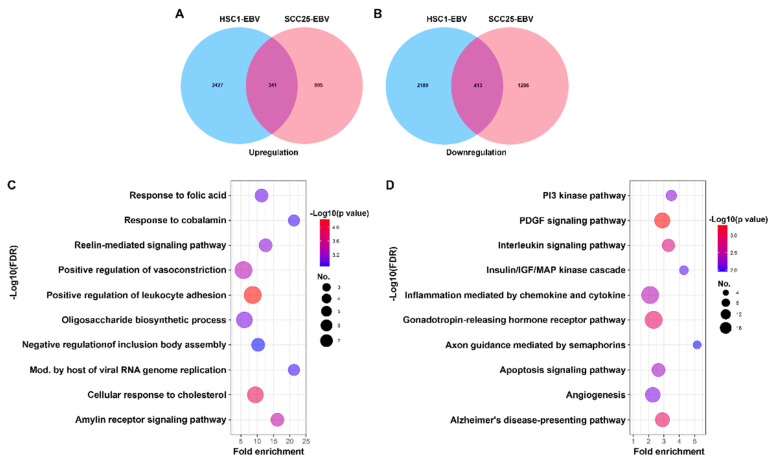
Common DEGs and functional annotation of DEGs in EBVaOSCC. The Venn diagram visualizes the upregulated (**A**) and downregulated (**B**) DEGs in SCC25-EBV and HSC1-EBV cells. Bubble diagram of functional enrichment analysis for DEGs. The top 10 enrichment results of the biological process (**C**) and KEGG pathway (**D**) are presented in a bubble diagram. The *Y*-axis on the left indicates the top 10 functional enrichment results. The *X*-axis indicates the percentage of genes involved in the biological process and the KEGG pathway. The color represents the *p*-value, and a range from red to blue indicates a low to high *p*-value, respectively. The size of the bubble indicates the gene numbers involved in the biological process and KEGG pathway.

Venn diagrams were constructed to screen the DEGs in EBV-associated OSCC (EBVaOSCC) cell lines, such as SCC25-EBV cells and HSC1-EBV cells. A total of 1190 DEGs with 341 upregulated and 413 downregulated genes were identified in EBVaOSCC cell lines (Figure 1A,B). To further understand the functional activity of DEGs, overlapping DEGs were submitted to the GO functional enrichment analysis. The top 10 enriched GO terms from the biological processes are shown in Figure 1C. In the biological process, the DEGs were significantly enriched in terms of positive regulation of leukocyte adhesion, cellular response to cholesterol, modulation by host of viral RNA genome replication, and so on (Figure 1C). For the KEGG analysis, the DEGs were significantly enriched in the pathways of the platelet derived growth factor (PDGF) signaling pathway, gonadotropin-releasing hormone receptor pathway, interleukin signaling pathway, and so on (Figure 1D).

**Figure 2:**
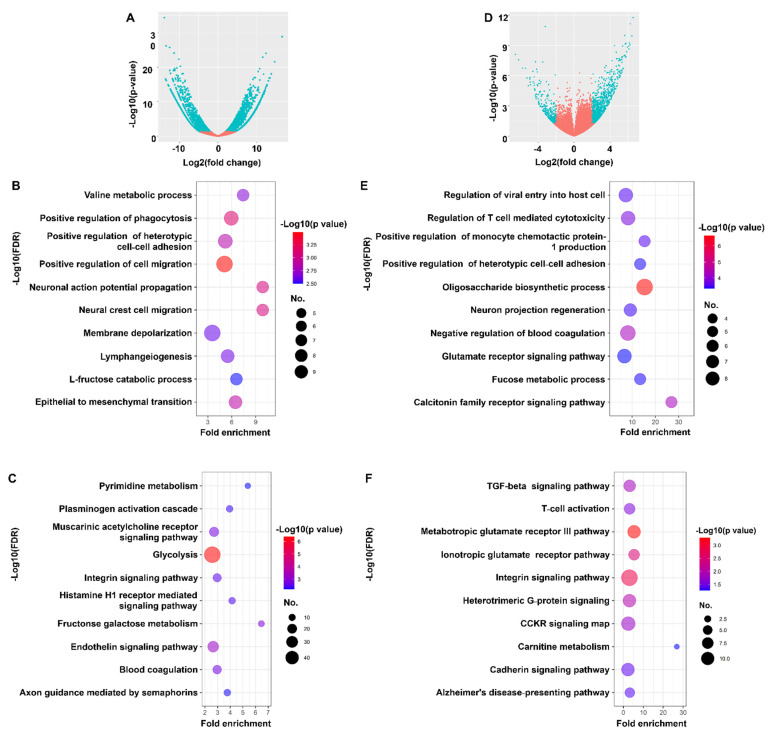
The characteristics and functional annotation of the DEGs in NPC and EBVaGC. The volcano plots illustrate the DEGs in NPC (**A**) and EBVaGC (**D**). The bubble diagram represents the result of functional enrichment analyses of DEGs of NPC and EBVaGC. (**B**,**C**) The top 10 enrichment results of the biological process and KEGG pathway in NPC and EBVaGC (**E**,**F**). The *Y*-axis on the left indicates the top 10 functional enrichment results. The *X*-axis indicates the percentage of genes involved in the biological processes and the KEGG pathway. The color represents the *p*-value, and a range from red to blue indicates a low to high *p*-value, respectively. The size of the bubble indicates the gene numbers involved in the biological process and KEGG pathway.

In this study, the RNA sequencing data of NPC and GC tumors were obtained from publicly available databases and further screened individually for DEGs. In terms of NPC, 2403 genes were differentially expressed between EBV-positive and EBV-negative cells. Among the 2403 genes, 1624 were upregulated, and 779 were downregulated in EBV-positive cells (Figure 2A). The GO terms of the DEGs are indicated in Figure 2B–D. The DEGs were mainly enriched in the positive regulation of cell migration, phagocytosis, epithelial-mesenchymal transition, and others (Figure 2B). In addition, the pathways that were enriched were the interleukin signaling pathway, endothelin signaling pathway, fructose galactose metabolism, and others (Figure 2C). Among the 1297 DEGs identified in EBVaGC cell lines, 978 were upregulated, and 319 were downregulated (Figure 2D). The GO terms of DEGs are indicated in Figure 2E,F. The DEGs were mainly enriched in the oligosaccharide biosynthesis process, calcitonin family receptor signaling pathway, negative regulation of blood coagulation, and others (Figure 2E). In addition, the enriched pathways were the metabotropic glutamate receptor group III pathway, interleukin signaling pathway, ionotropic glutamate receptor pathway, and others (Figure 2F). Therefore, these DEGs caused by EBV infection may provide new insights into the biological mechanisms of EBVaCAs and serve as potential therapeutic targets for EBVaCAs.

### 3.2. SLC26A9 and TMC8 as Promising Gene Signatures for EBVaCAs

**Figure 3:**
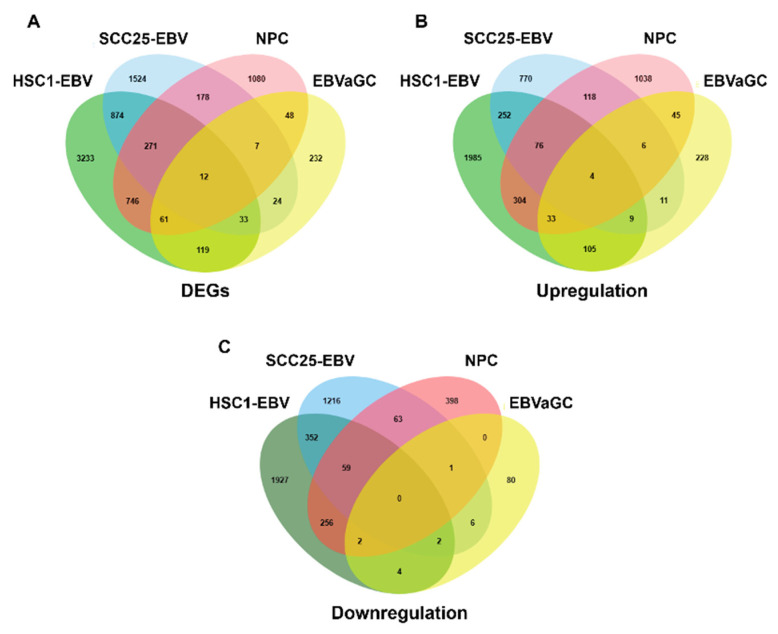
Differential expression of genes between EBV-infected OSCC, NPC, and GC cells. The Venn diagrams represent the DEGs (**A**), upregulated DEGs (**B**), and downregulated DEGs (**C**) in EBV-infected OSCC, NPC, and GC cells.

We further identified the signature gene of EBVaCAs using DEGs sets that were obtained from EBVaOSCC, NPC, and EBVaGC by constructing Venn diagrams. As shown in Figure 3A, 14 genes, including FUT4, S100A9, SCEL, BAMBI, ETV7, CHDH, CYP4F12, SLC26A9, SGPP2, TMC8, SDR16C5, and MCF2L-AS1, were differentially expressed in EBV-positive cancer cell lines. Four of the 14 genes, BAMBI, SLC26A9, SGPP2, and TMC8, were significantly upregulated in all EBV-positive cancer cell lines (Figure 3B). No downregulated genes were found in any of the EBV-positive cancer cell lines (Figure 3C).

**Figure 4:**
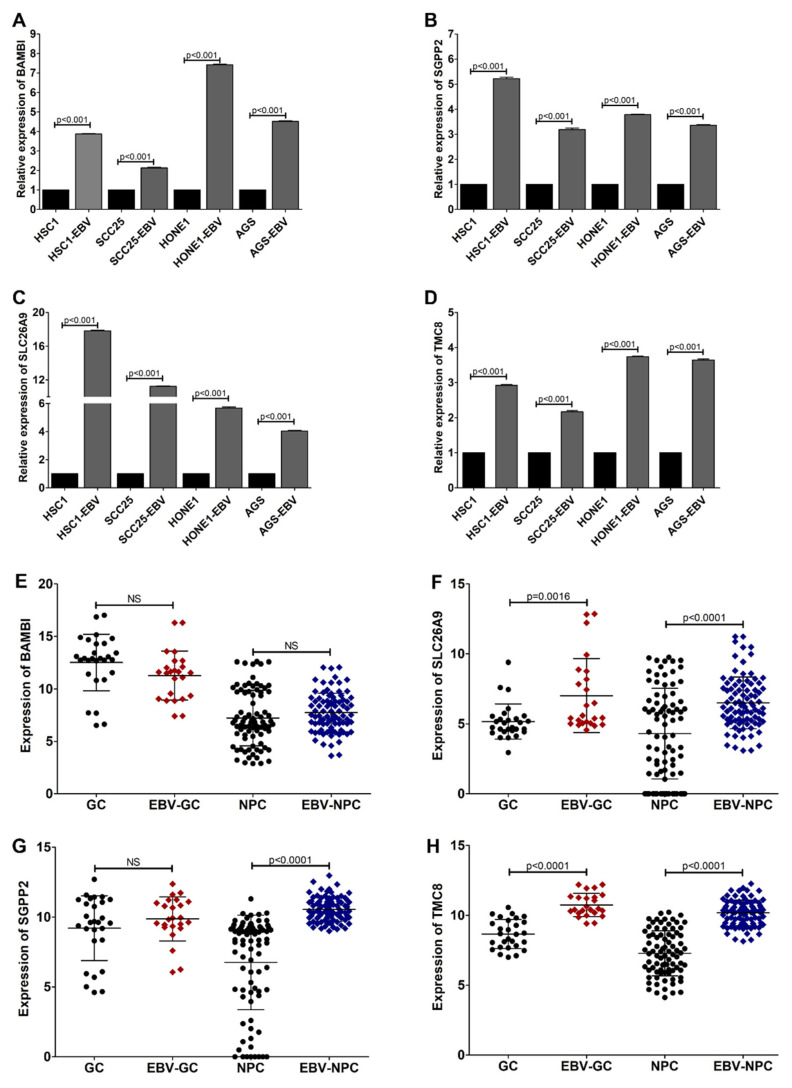
SLC26A9 and TMC8 could be used as a gene signature for EBV-associated epithelial cancers. The expression of BAMBI (**A**), SLC26A9 (**B**), SGPP2 (**C**), and TMC8 (**D**) was examined in both EBV-infected and EBV-uninfected cells by qRT-PCR. The expression of BAMBI (**E**), SLC26A9 (**F**), SGPP2 (**G**), and TMC8 (**H**) was also examined in tumor tissues of EBV-associated epithelial cancers.

Our bioinformatics analysis showed that the expression of BAMBI, SLC26A9, SGPP2, and TMC8 was significantly upregulated in EBV-positive cell lines compared with that in EBV-negative counterpart cell lines (Supplementary Figure S1). Therefore, we further confirmed whether the upregulated BAMBI, SLC26A9, SGPP2, and TMC8 genes could be used as a gene signature for EBVaCAs by determining the expression levels of these four genes in cell lines and tumor tissues by qRT-PCR. These four genes were significantly upregulated in AGS-EBV, HONE1-EBV, HSC1-EBV, and SCC25-EBV cells compared with that in individual EBV-negative counterpart cells (Figure 4A–D). Similarly, the expression of SLC26A9 and TMC8, but not BAMBI or SGPP2, was significantly upregulated in EBV-positive tumor tissues compared with that in EBV-negative tumor tissues (Figure 4E–H). These results suggest that the upregulation of SLC26A9 and TMC8 could be used as promising gene signatures for EBVaCAs.

### 3.3. TMC8 Is a Potential Gene Signature for EBV-Associated Malignancies

**Figure 5:**
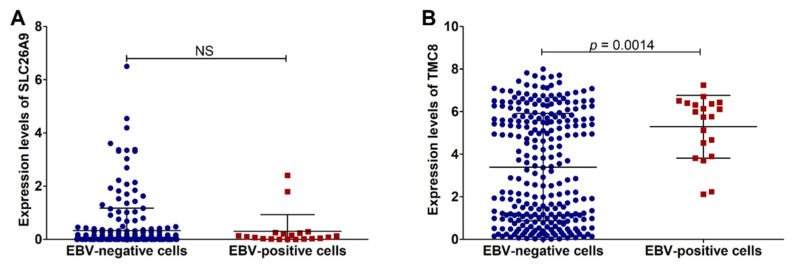
TMC8 is a potent signature gene for EBV-associated malignancies. The expression of SLC26A9 (**A**) and TMC8 (**B**) in cancer cell lines using RNA sequencing dataset from the CCLE database.

To identify our gene signature for EBV-associated malignancies, both lymphoma and epithelial malignancies, we further examined the expression of SCL26A9 and TMC8 in various types of cancer cell lines, including lymphoma and epithelial cancers, using the RNA sequencing dataset (DepMap 21Q4 Public) from the Cancer Cell Line Encyclopedia (CCLE) database. In the present study, 282 cell lines were included; among these, 20 cell lines were infected with EBV. As shown in Figure 5, the expression level of TMC8, but not SLC26A9, was significantly upregulated in EBV-positive cell lines compared with EBV-negative cell lines. Thus, this result suggests that TMC8 could be useful as a gene signature for EBV-positive malignancies.

### 3.4. EBV Induces IL6/JAK/STAT3 and TNF-α/NF-KB Signaling in EBVaCAs

**Figure 6:**
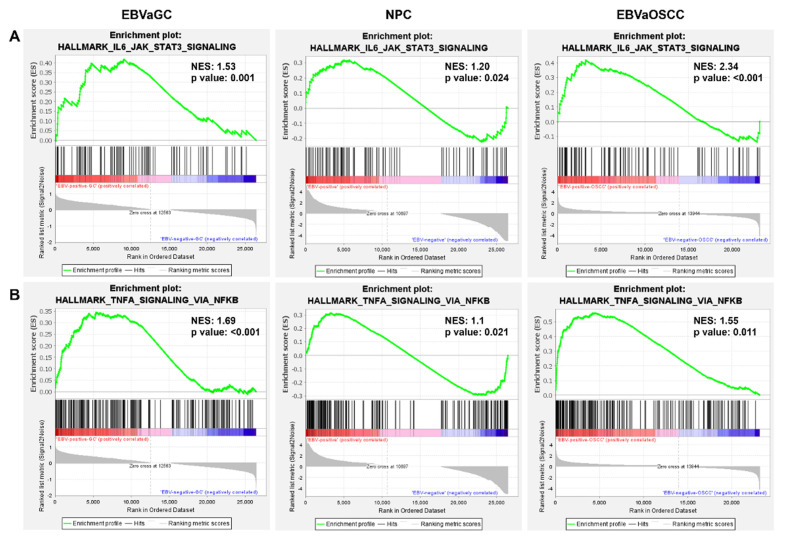
Identification of common hallmarks in EBV-associated epithelial cancers. Gene set enrichment analysis (GSEA) of genes in EBV-infected cells compared with non-EBV-infected cells. GSEA identified the IL6/JAK/STAT3 (**A**) and TNF-α/NF-κB (**B**) signaling pathways as a common hallmark in EBVaOSCC, NPC, and EBVaGC. The green line indicates enrichment profiles. The vertical black bar and the vertical grey bar indicate hits and ranking metric scores, respectively.

To better understand the underlying molecular mechanism by which EBV promotes the oncogenic process of EBVaCAs, GSEA analysis was performed to identify the hallmarks of DEGs in EBV-infected cells. The top five hallmarks of EBVaOSCC, NPC, and EBVaGC are listed in Table 2. The hallmark that was most enriched in EBVaOSCC was TNF-α/NF-κB signaling. Notch signaling was the most enriched hallmark of NPC. The IFN-α response was the greatest hallmark of EBVaGC. Furthermore, the common hallmarks of EBVaCAs were identified using GSEA. As expected, IL6/JAK/STAT3 signaling and TNF-α/NF-κB signaling were the two common hallmarks of EBVaCAs (Figure 6A,B). This result suggests that EBV mediates IL6/JAK/STAT3 signaling and TNF-α/NF-κB signaling to promote the carcinogenesis of EBVaCAs.

**Figure 7:**
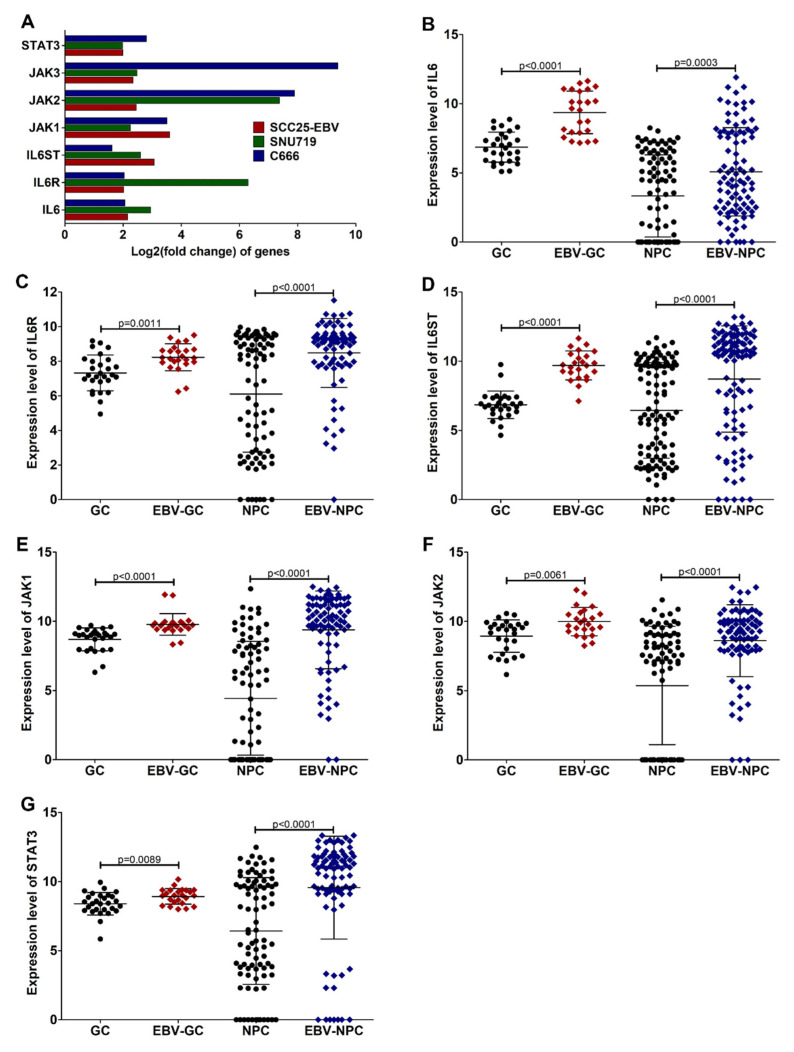
Key genes in the IL6/JAK/STAT3 signaling pathway are upregulated in EBV-associated epithelial cancers. The expression of key genes in the IL6/JAK/STAT3 signaling pathway was determined in EBV-infected cells (**A**). In the tumor tissues, the expression of IL6 (**B**), IL6R (**C**), IL6ST (**D**), JAK1 (**E**), JAK2 (**F**), and STAT3 (**G**) was also examined.

To confirm whether EBV induces IL6/JAK/STAT3 signaling and TNF-α/NF-κB signaling in EBV-associated cancers, the expression of key genes in these two common hallmarks was examined in the cell lines and tumor tissues of EBVaCAs. In accordance with the results shown in Figure 6, the expression of IL6, IL6R, IL6ST, JAK1, JAK2, and STAT3, which are key genes in the IL6/JAK/STAT3 signaling pathway, was significantly upregulated in EBV-positive cell lines, such as SCC25-EBV, SNU719, and C666-1 cells (Figure 7A). Similarly, the expression of these genes was also significantly higher in the EBV-positive tumor tissues than in the EBV-negative tumor tissues (Figure 7B–G).

**Figure 8:**
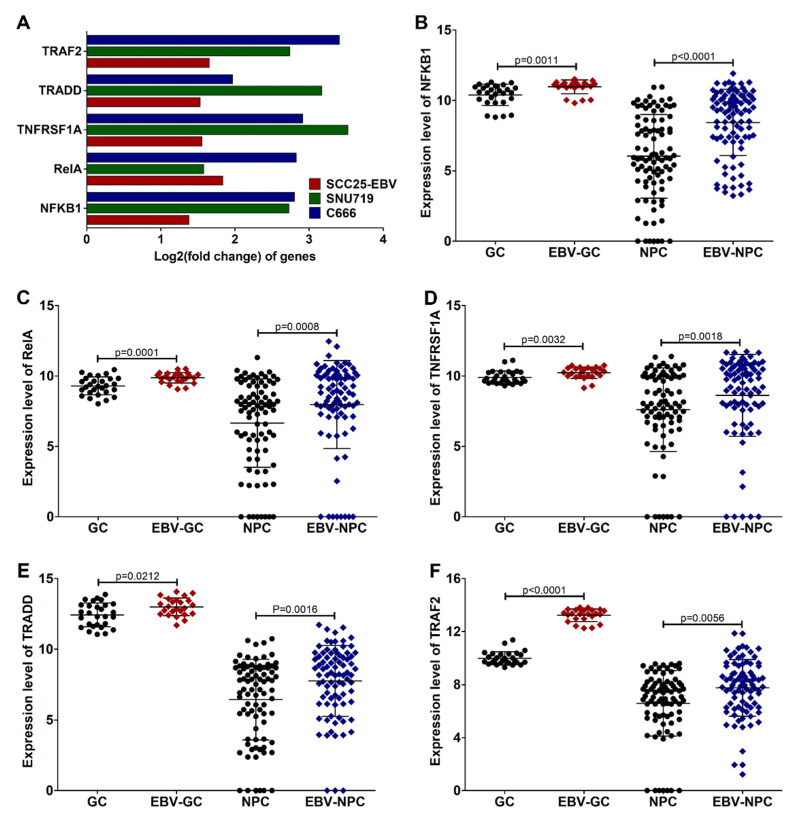
Key genes in the TNF-α/NF-κB signaling pathway are upregulated in EBV-associated epithelial cancers. The expression of key genes in the TNF-α/NF-κB signaling pathway was determined in EBV-infected cells (**A**). In the tumor tissues, the expression of NFKB1 (**B**), RelA (**C**), TNFRSF1A (**D**), TRADD (**E**), and TRAF2 (**F**) was also examined.

In addition, the expression of key genes in the TNF-α/NF-κB signaling, including NFKB1 (p50), RelA (p65), TNFRSF1A (TNFR1), TNFR1-associated death domain protein (TRADD), and TNF receptor-associated factor 2 (TRAF2), was examined in the cell lines and tumor tissues of EBV-associated cancers. As expected, the expression of key genes in the TNF-α/NF-κB signaling was significantly upregulated in the EBV-positive cell lines (Figure 8A). The expression of these genes was also higher in EBV-positive tumor tissues than in EBV-negative tumor tissues (Figure 8B–F).

## 4. Discussion

EBV is associated with various types of malignancies, including B-cell tumors and epithelial tumors, such as NPC and EBVaGC [3,32,33]. However, the genetic signature or biomarker characteristic of EBVaCAs has not been fully elucidated. Therefore, identification of the genetic signature is critical for the diagnosis, prognosis, and management of patients with EBV infection.

Previously, the differential expression of CRIP1, KITLG, MARK1, and PGAP1 was identified as a potential prognostic biomarker of NPC [34]. Ten immune-related genes were identified as prognostic biomarkers of GC [35]. The four antioxidant-related genes (CHAC1, GGT5, GPX8, and PXDN) were proposed as gene signatures to predict the prognosis of GC patients [36]. The five upregulated DEGs (HMMR, CCNB1, CXCL8, MAD2L1, and CCNA2) were proposed as potential biomarkers and therapeutic targets for GC treatment [37]. In addition to mRNA, five miRNAs, including let-7b-5p, miR-140-3p, miR-192-5p, miR-223-3p, and miR-24-3p, were significantly upregulated in the serum of patients with NPC and could be used as potential diagnostic biomarkers of NPC [38]. Similarly, the detection of seven miRNAs in plasma, let-7b-5p, miR-140-3p, miR-144-3p, miR-17-5p, miR-20a-5p, miR-20b-5p, and miR-205-5p, were mentioned as promising non-invasive diagnostic biomarkers of NPC [39].

We constructed a model of the gene signature for EBVaCAs at the transcriptomic level. Twelve genes were differentially expressed in EBV-positive cells. The dysregulated expression of these genes may be related to EBV infection. Two genes, SLC26A9 and TMC8, were identified as gene signatures for EBVaCAs because these two genes were upregulated in all EBV-positive cell lines and tumor tissues. Transcriptomics showed that the combination of SLC26A9 and the other four genes, SINHCAF, MICB, KRT19, and MT1X, became a gene signature that predicts the overall survival of esophageal adenocarcinoma [40]. The combination of SLC26A9 with AP002478.1, BHLHA15, FFAR2, IGFBP1, KCTD8, and PHYHD1 was also identified as a gene signature for personalized prognosis prediction and targeted therapy of esophageal adenocarcinoma [41]. SLC26A9 is a member of the solute-linked carrier 26 (SLC26) anion transporter family and is mainly expressed in epithelial cells. SLC26A9 functions uniquely as a chloride channel with minimal conductance to bicarbonate [32,33,34,35,36,37,38,39,40,41,42,43,44]. However, the role of SLC26A9 in cancer has not yet been elucidated.

TMC8, also known as EVER2, is a member of the mammalian transmembrane channel-like gene family [45]. TMC8 modulates the HPV life cycle by regulating the immune function [46]. The high expression of TMC8 may improve the prognosis of HNSCC because the upregulation of TMC8 is correlated with immune cell infiltration and the diversity of immune marker expression [47]. In addition, the combination of TMC8 with FRMD5, PCMT1, PDGFA, YIPF4, or ZNF324B has been proposed as a novel prognostic biomarker of HNSCC to guide the clinical treatment [48]. The ectopic expression of latent membrane protein 1, an oncogenic protein of EBV, inhibits the expression of TMC8 in B lymphocytes [49].

Although EBV infection promotes the carcinogenic process of EBVaCAs, the common mechanism of EBVaCA development by EBV infection is still unknown. Therefore, elucidating the molecular mechanisms underlying the carcinogenic process of EBVaCAs is important. In this study, we identified two common hallmarks of EBVaCAs, including the IL6/JAK/STAT3 and TNF-a/NF-κB signaling pathways. However, it remains unclear how these common hallmarks are activated in EBVaCAs.

The IL6/JAK/STAT3 pathway is hyperactivated in various types of cancer and is commonly associated with poor clinical prognosis. The IL6/JAK/STAT3 pathway plays an important role in the signal transduction processes, which are associated with cell proliferation and the invasive phenotype of cancers [50]. EBV infection activates the JAK/STAT3 pathway and promotes the proliferation and invasion of NPC cells [51]. The EBV lytic protein BRLF1 activates the IL6/JAK/STAT3 pathway and promotes cancer by inducing cell migration in NPC cells [52].

NF-κB plays a pivotal role in the regulation of cell survival, activation, and differentiation of innate immune cells and mediates inflammatory responses [53]. TNF-α is an inflammatory cytokine that plays an important role in the initiation of signal transduction by interacting with the TNF receptor (TNFR1). In the context of carcinogenesis, TNF-α plays a critical role in proliferation, migration, invasion, and angiogenesis. TNF-α also serves as a key regulator of the tumor microenvironment [54,55,56]. In EBVaCAs, EBV LMP1, a membrane protein that resembles the proteins in the TNF receptor superfamily, plays key roles in the activation of NF-κB by interacting with the TRAF and TRADD [57,58,59].

## 5. Conclusions

Based on the publicly available data and our experimental results, we demonstrate for the first time, the upregulation of SLC26A9 and TMC8 was identified as a gene signature for EBVaCAs by performing a comprehensive analysis of the mRNA profiles of EBVaCAs. The gene signature identified in this study may serve as a novel and reliable tool for predicting the patients’ prognosis and establishing the treatment strategies for EBVaCAs. This study also provides significant information for molecular research by identifying the common hallmarks of EBVaCAs, which are the IL6/JAK/STAT3 and TNF-a/NF-κB signaling pathways (Supplementary Figure S2).

## Figures and Tables

**Table 1:** Primer sequences.

Gene	Forward (5′-3′)	Reverse (5′-3′)
*BAMBI*	CCAAGGGAGCTGGAATTGAGT	ACTGTCTCACGTTTCCCAGTTA
*GAPDH*	TCATCAGCAATGCCTCCTGCA	TGGGTAGCAGTGATGGCA
*SGPP2*	ATACGGTCCTGGATGTGCTG	ATGACACACACGGGGAAGAG
*SLC26A9*	TTGCAAAAACCTCCCCCACA	TCTTGTGCATGTAGCGAGCA
*TMC8*	AGGAGTCGTCTGAGAAGGGG	GAGAGAATCCTGCTGCGGTC

**Table 2:** Top 5 hallmarks in EBVaOSCC, NPC, and EBVaGC.

Hallmark	NES	NOM *p* Value	FDR q Value
**EBVaOSCC**			
HALLMARK_TNFA_SIGNALING_VIA_NFKB	2.34	<0.001	<0.001
HALLMARK_HYPOXIA	1.85	<0.001	0.002
HALLMARK_UV_RESPONSE_UP	1.81	<0.001	0.003
HALLMARK_P53_PATHWAY	1.73	<0.001	0.009
HALLMARK_REACTIVE_OXYGEN_SPECIES_PATHWAY	1.7	0.01	0.008
**NPC**			
HALLMARK_NOTCH_SIGNALING	1.27	0.12	0.615
HALLMARK_KRAS_SIGNALING_DN	1.22	0.02	0.451
HALLMARK_TNFA_SIGNALING_VIA_NFKB	1.2	0.024	0.37
HALLMARK_UV_RESPONSE_UP	1.19	0.037	0.299
HALLMARK_IL6_JAK_STAT3_SIGNALING	1.1	0.021	0.485
**EBVaGC**			
HALLMARK_INTERFERON_ALPHA_RESPONSE	2.57	<0.001	<0.001
HALLMARK_INTERFERON_GAMMA_RESPONSE	2.33	<0.001	<0.001
HALLMARK_XENOBIOTIC_METABOLISM	1.79	<0.001	0.003
HALLMARK_INFLAMMATORY_RESPONSE	1.76	<0.001	0.004
HALLMARK_IL6_JAK_STAT3_SIGNALING	1.69	<0.001	0.007

EBVaOSCC: Epstein-Barr virus˗associated oral squamous cell carcinoma, NPC: nasopharyngeal carcinoma (NPC) and EBVaGC: Epstein-Barr virus˗associated gastric carcinoma (EBVaGC).

## Data Availability

The published articles included all datasets generated or analyzed during this study.

## Supplementary Materials

The following are available online at https://www.mdpi.com/article/10.3390/cancers14010031/s1, Figure S1: The expression of candidate genes in EBVaCAs cells, Figure S2: The schematic representation of the study design and findings of this study.
