# Supplementary material for: General Features and Novel Gene Signatures That Identify Epstein-Barr Virus-Associated Epithelial Cancers
Source: Cancers (Basel). 2021 Dec 22;14(1):31. doi: 10.3390/cancers14010031 (PMC8750470; doi:10.3390/cancers14010031)
Supplement: Supplementary file 1 [file cancers-14-00031-s001.zip › cancers-1486760-supplementary.pdf]

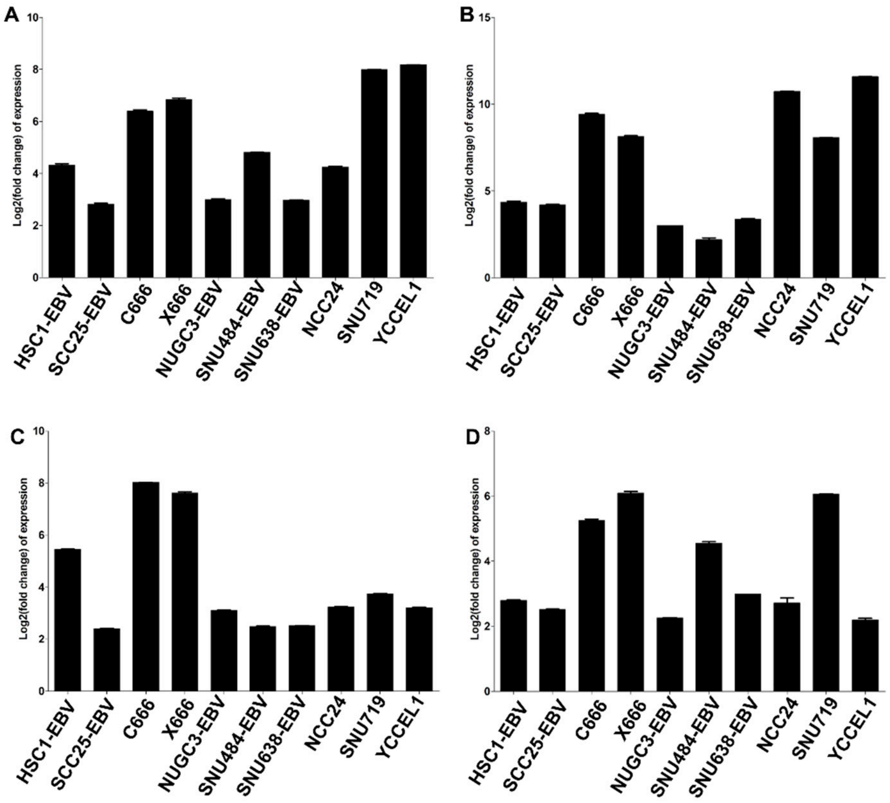

Figure S1. The expression of candidate genes in EBVaCAs cells. The expression of BAMBI (**A**), SGPP2 (**B**), SLC26L9 (**C**), and TMC8 (**D**) was determined by transcriptomics approaches.

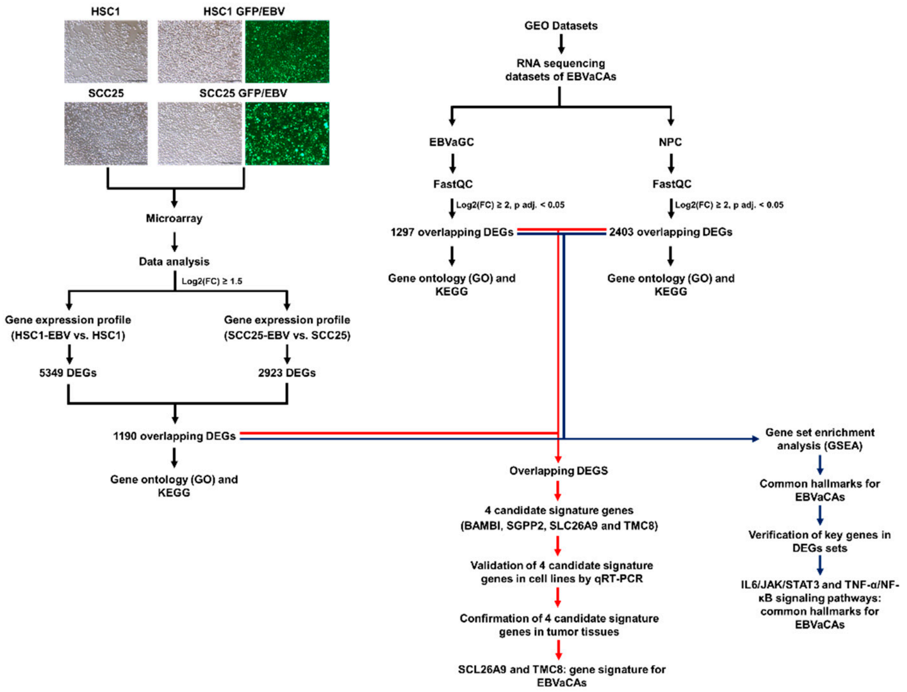

Figure S2. The schematic representation of the study design and findings of this study.
